# Nutritional Values and Biochemical Traits of Rye (*Secale cereale* L.) Seeds, a Landrace from Matese Mountains (Southern Italy)

**DOI:** 10.3390/foods14071120

**Published:** 2025-03-24

**Authors:** Nicola Landi, Sara Ragucci, Maria Giuseppina Campanile, Hafiza Z. F. Hussain, Stefania Papa, Antimo Di Maro

**Affiliations:** 1 Institute of Crystallography, National Research Council, Via Vivaldi 43, 81100 Caserta, Italy; 2 Department of Environmental, Biological and Pharmaceutical Sciences and Technologies (DiSTABiF), University of Campania ‘Luigi Vanvitelli’, Via Vivaldi 43, 81100 Caserta, Italy; sara.ragucci@unicampania.it (S.R.); mariagiuseppina.campanile@unicampania.it (M.G.C.); hafizazumrafatima.hussain@unicampania.it (H.Z.F.H.); stefania.papa@unicampania.it (S.P.)

**Keywords:** amino acids, *Secale cereale* L., food quality, Matese mountains, metabolic profile, proximate composition

## Abstract

Rye (*Secale cereale* L.) from Matese mountains is a local landrace cultivated in Southern Italy. To promote the benefits for the mountain economy, we report on the nutritional values (crude proteins, lipids, moisture, ash and total amino acids), metabolic traits (free amino acids, fatty acids and bioactive organic compounds) and mineral content of ‘segale del Matese’ seeds. Seeds were collected in 2023 and 2024 at two altitudes (~1000 and 150 m a.s.l.); these were analyzed, and the results were compared. Average data from two sites (crude proteins (9.6 g/100 g), lipids (1.3 g/100 g), ash (1.8 g/100 g), moisture (9.3 g/100 g) and carbohydrates (78.3 g/100 g)) show few significant statistical differences. The same trend was observed for total amino acid content, except for Glx (glutamic and glutamine), while statistical differences between the two sites were found among proteinogenic free amino acids. Moreover, segale del Matese’ is rich in polyunsaturated (linoleic and linolenic) and monounsaturated (oleic) fatty acids. Furthermore, total phenolic content, some bioactive compounds (i.e., gallic acid, vanillic acid, *p*-coumaric acid and ferulic acid) and radical scavenging activity were estimated. Finally, the seeds subjected to thermal treatment showed a decrease in anti-trypsin and anti-chymotrypsin activities, resulting in a favorable depletion of antinutritional factors.

## 1. Introduction

Rye (*Secale cereale* L.) is an ancient cereal of the Poaceae family, which has been cultivated since the Bronze Age (3000–1200 BC) in Asia Minor [1,2]. Rye is a hardy (resilient) cereal that can withstand extreme cold, and it has lower heat requirements and a shorter growth cycle than other cereals. In light of this, the cultivation of rye is common in those areas where agriculture is difficult and marginal (e.g., mountain areas), even on acidic, sandy and poor soils [2]. Indeed, the largest producers are mainly located in the cold regions of the northern hemisphere (i.e., Germany, Poland, Russia and China) at altitudes ≥ 800 m a.s.l. [3].

Rye seeds are refined into flour, traditionally used alone or in combination with other flours (e.g., wheat) for the preparation of brown bread, pasta, snacks, biscuits and breakfast cereals [4,5]. The proximate composition of rye, expressed as a percentage of dry matter, includes carbohydrates (56–70%), crude proteins (8–13%), lipids (2–3%), ash (2%) and total dietary fibers (15–21%) [5,6]. Compared to wheat, rye contains 15–21% more dietary fibers, of which 20% are soluble, including arabinoxylans (8–12%) with prebiotic activities [7], β-glucans (1.3–2.2%), known as potent immunomodulators with anticancer properties [8], and cellulose (1–1.7%). On the other hand, rye flour has a lower crude protein content with respect to wheat flour, and, like other cereals, it contains gluten. However, considering the crude proteins, rye flour has a well-balanced amino acid profile, with a higher lysine content, which is well known to be lower in all cereals [9]. In addition, rye flour is a rich source of bioactive compounds (alkylresorcinols, ferulic acid, catechol, sinapic acid, vanillin and vanillic acid) with antioxidant potential [10] and micronutrients, making it a nutraceutical food for its health benefits [11]. In particular, micronutrients like vitamins (riboflavin, tocopherol, thiamine, B6, folate, niacin and choline) [11] and minerals such as potassium, magnesium, calcium, zinc and iron [12] are present in rye flour.

Modern consumers pursuing a healthy lifestyle, oriented toward the prevention of health problems, consider this product as a part of their diet since its consumption can lead to the following benefits: (i) a reduction in the risk of type 2 diabetes [13]; (ii) a cholesterol-lowering effect [14]; (iii) an improvement in colon function with a reduction in the concentration of some molecular biomarkers which increase the risk of colon cancer [9,15]. Therefore, considering the positive impact of rye-based products on human health in chronic and inflammatory human diseases, the use of rye flour is increasing [16,17].

The cultivation of this cereal in Italy is distributed in the mountainous areas of the Alps and the Apennines, although this trend is decreasing due to the depopulation of marginal mountain areas [18]. In Italy, the surface area planted with rye amounts to approximately 3340 hectares, mainly distributed in marginal and mountainous areas of Southern Italy, with production resulting in 10,500 tonnes per year [19]. In mountainous areas, bread made from rye flour has been the mainstay of local populations during times of war or famine. Several agronomic and genetic studies on rye have shown that different landraces are present in Italy, due to specific pedoclimatic conditions [20,21]. Landraces are genetically heterogeneous plant populations utilized in subsistence agriculture, which had adapted to the specific pedoclimatic conditions of the territory in which they have evolved [22].

In this framework, ‘segale del Matese’ (‘sécena’ in the local dialect) is a local landrace recognized to be a typical product of the Campania Region (Southern Italy). This landrace shows dressed and greyish caryopses, typically elongated with pointed apices. Flour obtained from this rye is cultivated in the towns of S. Gregorio Matese, Gallo Matese and Letino (Matese plateau, province of Caserta, Campania region) and has been used for centuries to make brown bread [23].

Considering the above context, the present investigation aims to establish, for the first time, the nutritional values (crude proteins, lipids, ash, moisture and carbohydrates) and biochemical traits (total and free amino acids, fatty acids and antioxidant capacity and some bioactive organic compounds) of ‘segale del Matese’ flour obtained from rye cultivated in Matese mountains, collecting the seeds for two consecutive years (2023 and 2024). In addition, considering the antinutritional factors (e.g., trypsin and chymotrypsin inhibitors), we analysed the presence of these protease inhibitors in raw and boiled rye seeds.

In addition, the same analyses were performed on ‘segale del Matese’ flour obtained from seeds grown in the experimental fields of Agrarian Institute of Piedimonte Matese (province of Caserta), with the intention of promoting the local marketing of rye brown bread. This parallel study allowed us to verify a possible variation in the nutritional values and biochemical traits of ‘segale del Matese’ flour, related to the different areas of cultivation, considering that the seeds used in these experimental fields were donated by farmers of Letino and Castello del Matese municipalities.

## 2. Materials and Methods

### 2.1. Chemicals and Reagents

Folin–Ciocalteu reagent, gallic acid, 2,2′-azino-bis(3-ethylbenzothiazoline-6-sulphonic acid) (ABTS), p-toluenesulfonyl-L-arginine methyl ester (TAME), N-benzoyl-L-Tyrosine ethyl ester (BTEE) and *nor*-leucine (*nor*-leu) were obtained from Sigma-Aldrich Solutions (Merck Life Science S.r.l., Milan, Italy). Chemicals and solvents for the Kjeldahl method and automated amino acid analysis were provided by Carlo Erba Reagents Srl (Cornaredo, Milan, Italy) and Errecci Srl (Opera, Milan, Italy.), respectively. All other reagents and chemicals were of analytical grade and have been described previously [24,25].

More details for specific reagents or enzymes are reported below in their relevant paragraphs.

### 2.2. Plant Material and Sampling

*Secale cereale* (SC) was grown in 2023 and 2024 by the ‘Falode’ farm (https://www.falode.it/; accessed on 7 January 2025) and the Agricultural Technical Institute of Piedimonte Matese (https://www.isissmatese.edu.it/; accessed on 7 January 2025). The respective fields are located in the municipalities of Castello del Matese (coordinates: 41°24′12.9″ N 14°26′17.6″ E; altitude: 1020 m), hereafter site 1(s1), and Alife (coordinates: 41°20′24.3″ N 14°19′42.2″ E; altitude: 130 m), hereafter site 2(s2). Rye seeds of ‘segale del Matese’ used for the sowing were donated by the farmers of Letino and Castello del Matese municipalities. Following the harvest, for each year and site, three different pools of ‘segale del Matese’ were sampled and called SC-s1 and SC-s2, according to the respective field sites. Subsequently, the seeds were cleaned to remove any impurities, and sundried until the humidity reached approximately 12%. In order to obtain rye flour, seeds were ground to a fine powder by using Cyclone Sample Mill (Model 3010-019, PBI International, Milan, Italy) equipped with a 0.25 mm screen, and stored at −80 °C for future analyses.

### 2.3. Proximate Composition

The macronutrient content of rye flour was determined according to the AOAC official method [26]. Crude protein content was obtained by the Kjeldahl method (AOAC 920.87; nitrogen factor used 5.83), while total lipid by using Soxhlet apparatus using CHCl_3_ as extracting solvent (AOAC 948.22). Total carbohydrate content was obtained by subtracting the value of total ash, lipids and proteins from the total dry matter, following the FAO indications [27].

### 2.4. Ash Content and Moisture Content

Ash content and moisture level were determined according to the AOAC official method [26]. In particular, the AOAC methods for ash and moisture content were AOAC 923.03 and AOAC 925.10, respectively.

### 2.5. Amino Acid Composition

In order to evaluate the total (free and protein) amino acids composition, aliquots of ~10 mg of rye flour were hydrolyzed at 110 °C for 20 h in 0.5 mL of 6.0 M HCl containing 0.02% phenol and *nor*-Leu as an internal standard. Following the hydrolysis, HCl was removed under vacuum and the hydrolysate was resuspended in 0.5 mL of 0.2 M Li–citrate buffer, pH 2.2.

In order to evaluate the free amino acid composition, aliquots of ~200 mg of rye flour were subjected to ethanol extraction using 80% cold ethanol (1.0 mL) in the presence of *nor*-Leu (50.0 nmol), homogenized with a teflon pestle and centrifuged at 14,000× *g*, 4 °C. The supernatant was lyophilized, and treated with 3.0% sulfosalicylic acid (500 µL) at 4 °C to precipitate any protein fraction still present [28]. The supernatant obtained after centrifugation (14,000× *g*, 4 °C) was directly analyzed. Aliquots of hydrolyzed and non-hydrolyzed samples were directly analyzed on a Biochrom 30 amino acid analyzer (Biochrom, Cambridge, UK), equipped with a post-column ninhydrin derivatization system, adapting a previously reported procedure [29].

### 2.6. Determination of Trypsin and Chymotrypsin Inhibitory Activities

Three replicate samples of rye flour (1.0 g each) were extracted overnight under magnetic stirring at 4 °C in 1× phosphate-buffered saline (PBS, 1:4; *w*/*v*). The mixtures were centrifuged at 24,000× *g* (Avant J-25, Beckman Coulter, Brea, CA, USA), 4 °C, for 60 min. The supernatants were filtered on Miracloth (pore size: 22–25 µm; VWR International Srl, Milan, Italy) and then aliquoted and stored at −80 °C until further analyses. In addition, to evaluate the effect of cooking process on the protease-inhibiting capacity, rye seeds (5.0 g) were soaked in water (1:5; *w*/*v*) for 12 h. Following this step, samples were boiled for 60 min in 150 mL water. Subsequently, proteins from both cooked and drained rye seeds were extracted, as described above. Protein concentration was determined with the Bio-Rad Protein Assay kit following the manufacturer’s instructions and using bovine serum albumin (BSA) as a standard.

Trypsin and anti-trypsin activities, as well as chymotrypsin and anti-chymotrypsin activities, were determined following a procedure already described [30], using TAME and BTEE, respectively, as substrates [31]. An inhibitory unit is defined as the amount of inhibitor producing a 30% decrease in enzyme activity under the assayed experimental conditions [31]. An increasing concentration of rye seeds protein extract was added to a fixed concentration of protease, which, in our conditions, corresponded to 0.05 absorbance/min, by monitoring the reaction for 5 min. Residual enzyme activities are expressed as a percentage of the control samples (enzyme activity in the absence of protein extract). The IC_50_ (half-maximal inhibitory concentration) values of trypsin and α-chymotrypsin activities from rye seeds protein extract were calculated as previously reported and expressed as µg of protein per mL of extract [30].

### 2.7. Gas Chromatographic Analysis of Fatty Acid Methyl Esters

The analyses of fatty acid methyl ester content were performed as previously reported [32]. Briefly, each crude lipid extract (see Section 2.3) was dissolved in 2.0 M KOH in methanol (0.2 mL). The solution was stirred for 30 min at 25 °C, mixed with heptane (0.8 mL) and then centrifuged at 4200× *g* for 10 min at 4 °C [Beckman GS-15R (Beckman Coulter, CA, USA)]. The upper organic phase (1.0 µL) was analyzed by GC column (15 m × 0.25 mm i. d., 0.2 µm SP2380, fused silica capillary column; Supelco, Sigma-Aldrich, St. Louis, MO, USA) by using a TRACE^TM^ 1310 gas chromatograph with a flame ionization detector (FID) (Thermo Fisher Scientific, Rodano, Milan, Italy). The fatty acid methyl esters were identified by comparing their retention times with those of the standard fatty acid methyl ester (Supelco^TM^ 37 Component FAME Mix; Sigma-Aldrich, St. Louis, MO, USA) and quantified using 50 µg of nonadecanoic acid as an internal standard. Each sample was prepared individually and analyzed in triplicate.

### 2.8. Extraction of Free Phenolics

Free phenolics were extracted according to Irakli et al., 2012, with the same modifications [33]. Briefly, 1.0 g of rye flour was resuspended in 3.0 mL of methanol/water (70:30; *v*:*v*) and sonicated at 30 °C for 15 min. The extract obtained was centrifuged at 10,000× *g* for 10 min at 25 °C (Allegra V-15R (Beckman Coulter, CA, USA)). The supernatant was collected, and the extraction was repeated in the same way. The extracts were pooled and stored at −80 °C for further analysis.

### 2.9. Determination of Phenolics Content and Antioxidant Evaluation

Total free phenolic content (TPC) in the methanolic extract (see Section 2.8) was determined by using the Folin–Ciocalteu method. TPC value was expressed as mg of gallic acid equivalents (GAE) per 100 g of seed powder [34].

ABTS^•+^ radical cation scavenging capacity of methanolic extract (see Section 2.8) was estimated as previously reported [34]. The results were expressed as mmol Trolox^®^ Equivalents Antioxidant Capacity (TEAC) per 100 g of seeds.

### 2.10. Identification and Quantification of Chemical Compounds Using RP-HPLC

The free polyphenol extract was characterized by RP-HPLC, as previously reported [33]. Briefly, the methanolic extract (see Section 2.8) was evaporated to approximately 1.0 mL at 30 °C using refrigerated centrifugal vacuum concentrator (SpeedVac™ SRF110 (Thermo Fisher Scientific, Waltham, MA, USA)). The concentrated extract was then mixed with 2.0 mL water containing 1.0% acetic acid. Acidified extract was then centrifuged at 10,000× *g* for 15 min at 25 °C (Allegra V-15R (Beckman Coulter, CA, USA)) and loaded onto a Sep-Pak^®^ C18 cartridge (Waters S.p.A., Sesto San Giovanni (MI, Italy)). The cartridge was then washed with 3.0 mL water to remove sugars and other polar constituents, and the absorbed compounds were subsequently eluted with 2.0 mL methanol/acidified water (9:1; *v*:*v*). Finally, the eluted samples were evaporated to dryness at 30 °C using a SpeedVac™ and resuspended in 200 µL methanol/acidified water (1:9; *v*:*v*). Samples were then analyzed by RP-HPLC using a Waters system equipped with a binary pump and a multi-wavelength UV/visible detector. Briefly, samples were loaded onto an Ultrasphere ODS (C18) column ((250 × 4.6 mm, 5.0 µm particle size; (Beckman, CA, USA)) at 25 °C, equilibrated with 10% solvent B (methanol) over solvent A (Milli-Q water containing 1.0% acetic acid). Aliquots of free polyphenol extract (100 µL) were eluted at flow rate of 1.0 mL/min using the following gradient: 10% B for 5 min, 20% B for 10 min, 25% B for 10 min, 35% B for 10 min, 65% B for 10 min and 90% B for 5 min. The main polyphenols were identified based on the retention times of authentic standard references, monitoring the absorbance at 270 and 320 nm. The standard used as a reference were as follows: gallic acid (1), protocatechuic acid (2), 4-hydroxybenzoic acid (3), vanillic acid (4), caffeic acid (5), p-coumaric acid (6) and ferulic acid (7). Stock solutions (1.0 mg/mL) of the standards were prepared by dissolving the appropriate amount of each standard in methanol and stored at −20 °C. The calibration curve was established using different quantities of each standard in a range of 1.25–5.0 µg. The final injection volume for both standards and samples was 1.0 mL dissolved in solvent A.

### 2.11. Determination of Minerals Content

Aliquot of the powdered samples (~250 mg) were mineralized in an Ethos 900 microwave digestion system (Milestone™ Srl, Sorisole, Bergamo, Italy) endowed with temperature control, by a combination of hydrogen peroxide and nitric acid (H_2_O_2_ 50% *v*/*v*: HNO_3_ 65% *v*/*v* = 1:3). After digestion, the solutions were diluted by deionized water to a final volume of 50 mL. The nutrient (Ca, Cu, Fe, K, Mg, Mn, Na, P, Se and Zn) concentrations were quantified by atomic absorption spectrometry (SpectrAA 20 Varian, Mulgrave, Victoria, Australia) via flame furnace using standard solutions (STD Analyticals, Carlo Erba, Val de Reuil, France). Accuracy was checked by analysis of standards (Resource Technology Corporation, Laramie, WY, USA) and the recovery was in a range of 90–110% for each element.

### 2.12. Statistical Analysis

Analyses were repeated three times for each sample; the means and standard deviations (SDs) of the experimental values are reported. Data analysis was carried out with Excel Microsoft 365 (Microsoft Corporation, Redmond, WA, USA). The IC_50_ values were calculated based on inhibition curves by plotting the residual enzyme activities versus different concentrations of protein extract by fitting data with a non-linear regression analysis on a semi-logarithmic scale by using the GraphPad Prism 8 software (GraphPad Software Inc., Boston, MA, USA). The results were statistically analyzed using two-way ANOVA test followed by Tukey’s and Bonferroni’s Multiple Comparison tests. The significance was accepted for *p* < 0.05.

## 3. Results and Discussion

### 3.1. Nutritional Values

The proximate compositions of ‘segale del Matese’, obtained by analyzing two different sites (SC-s1 and SC-s2) for each harvest year (2023 and 2024), are shown in Table S1. The analysis of crude protein, moisture and carbohydrates shows statistical differences, which are likely due to the different altitude of the two sites, as well as the climatic trends and variability of the two harvest years considered. The average values of two different ‘segale del Matese’ samples were then compared with those of commercial rye, as reported by the CREA database (see Table 1).

Average data from two cultivation sites did not show any significant difference. The protein content of ‘segale del Matese’ (9.6 g per 100 g of rye seeds) is in agreement with Hansen et al. (2004), who reported a protein content ranging from 8.0 to 11.3 g per 100 g of rye seeds, considering different rye varieties analyzed [6], while it was about 1.2-fold lower than that reported by CREA database. Furthermore, the lipid content of ‘segale del Matese’ was about 1.3 g per 100 g of rye seeds and 1.5-fold lower than that reported by the CREA database. In addition, the carbohydrate content of ‘segale del Matese’ (78.27 g per 100 g of rye seeds) was about 1.2-fold higher than the value reported by the CREA database. On the other hand, a similar moisture content was observed for all rye varieties considered. Finally, the ash content of ‘segale del Matese’ (1.8 g per 100 g of rye seeds) was in good agreement with Hansen et al. (2004), who found an ash content ranging from 1.8 to 2.2 g per 100 g of rye seeds, considering 17 varieties analyzed [6].

Overall, the consumption of 100 g of ‘segale del Matese’ can provide a caloric intake of about 362 Kcal (~78% carbohydrates, ~9.6% proteins and ~1.3% lipids), capable to cover about 14% or 18% of the average energy requirement for an adult male or female, respectively.

### 3.2. Amino Acid Content

Protein quality is based on the amino acid composition and content, as well as the availability of essential amino acids. Therefore, in order to evaluate the protein quality of rye seeds, total amino acid composition (free plus protein) of ‘segale del Matese’ was determined by subjecting rye seeds flour to acid hydrolysis, samples from two different sites and harvest years. As shown in Table S2, significant differences in the amino acid composition of two different ‘segale del Matese’ samples were found. Subsequently, the average values of two different ‘segale del Matese’ samples were compared with those of commercial rye, as reported by CREA database [35] (see Table 2).

These data showed no significant differences between ‘SC-s1’ and ‘SC-s2’ samples, except for Glx (glutamic acid + glutamine) content, using Bonferroni’s multiple comparison test. Specifically, ‘SC-s1’ has a Glx content 1.11-fold higher than ‘SC-s2’. Furthermore, Glx was the most abundant amino acid in ‘segale del Matese’ samples, with a percentage value of about 22% of total amino acids, this is in good agreement with that reported for commercial rye (~23% of total amino acids). Furthermore, the analyses performed showed that, among non-essential amino acids, the most abundant after Glx was proline, with an average value of about 0.80 g per 100 g, representing about 11% of average total amino acid content. This percentage content was about 1.8-fold higher than the percentage content of commercial rye. These amino acids were followed by Asx (aspartic acid + asparagine), with an average percentage of 6.3% of total amino acid content, which was about 1.3-fold lower than the percentage of Asx retrieved in commercial rye. In addition, the average total amount of essential amino acids in ‘segale del Matese’ was 2.4 g per 100 g and represented about 33% of the total amino acid composition, differently from the commercial rye with about 37% of essential amino acids. On the other hand, when the percentage of each essential amino acid was considered, the values of ‘segale del Matese’ and the commercial rye are similar. In particular, leucine, phenylalanine, lysine and valine were found to be the most abundant essential amino acids, with average percentages ranging from 7.0 to 4.0% of the total amino acids.

Furthermore, the free amino acid content of ‘segale del Matese’ samples is averagely 2.7% of the total amino acid composition (see Table S3). In addition, the statistical analysis revealed significant differences between the ‘SC-s1’ and ‘SC-s2’ samples, most likely due to the different altitudes of two fields and the two harvest years. These differences were found only in the proteinogenic free amino acids, while non-proteinogenic free amino acids are comparable.

In this framework, our data highlight that the most abundant free amino acid in ‘segale del Matese’ seeds was asparagine with an average value of 63 mg and 54 mg per 100 g of rye seeds, for SC-s1 and SC-s2, respectively. This amino acid plays a crucial role in the storage and transport of nitrogen in plants, and it is also the main contributor to the formation of acrylamide during the cooking of rye-based goods [36]. On the other hand, a comparison of ‘segale del Matese’ average free asparagine value of ~4.0 mmole per kg of rye seeds (~58.5 mg per 100 g) with the values of other 12 rye varieties, analyzed by Curtis et al. in 2010, highlighted that ‘segale del Matese’ has a asparagine content ranging from 1.15- to 2.04-fold lower, comparable to that of the Amilo rye variety (3.9 mmole per kg of rye seeds) [37]. This is of interest, considering that the lower free asparagine content of ‘segale del Matese’ with respect to the other varieties may lead to the production of less acrylamide during baking. In addition, non-proteinogenic free amino acids on average represent only 5.8% of total free amino acids for all samples analyzed, and GABA was the most abundant, with an average value of 4.4 mg per 100 g of rye seeds, corresponding to ~40% of all non-proteinogenic free amino acids.

**Figure 1:**
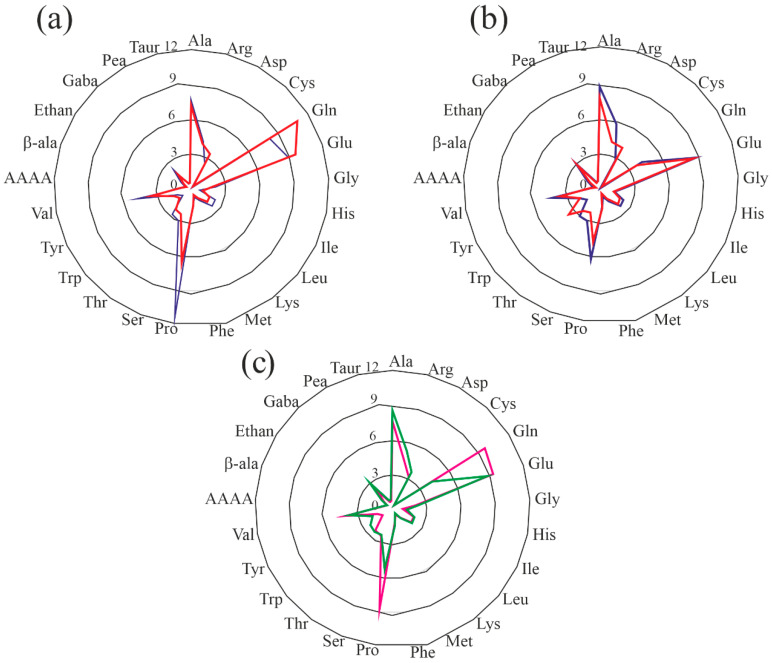
Radar graph of free amino acids average percentage. (**a**) Free amino acids percentage of *S. cereale* site 1 (SC-s1) for two consecutive harvest years (2023 and 2024, blue and red, respectively). (**b**) Free amino acids percentage of *S. cereale* site 2 (SC-s2) for two consecutive harvest years (2023 and 2024, blue and red, respectively). (**c**) Average percentage of free amino acids of SC-s1 and SC-s2 samples (pink and green, respectively).

Furthermore, in order to establish a possible relationship between the free amino acid profiles of ‘segale del Matese’ samples (‘SC-s1’ and ‘SC-s2’, harvested year 2023 and 2024), the percentages of free amino acids (except for asparagine content) were compared, as shown in Figure 1a–c.

The radar graphs show a similar trend between two harvested years for both samples, highlighting only some quantitative differences. In particular, for ‘SC-s1’ sample (see Figure 1a), the amount of proline, valine, isoleucine and leucine was, respectively, ~1.75, 1.37-, 1.32- and 1.31-fold higher in the 2023 samples with respect to the 2024 samples. On the other hand, the amount of glutamine and glutamic acid was, respectively, ~1.35- and 1.08-fold higher in the 2024 samples with respect to the 2023 samples. Furthermore, the same trend can be observed for the SC-s2 sample (see Figure 1b), since the 2023 sample showed contents of arginine, proline, valine and alanine, respectively, that were ~1.38-, 1.33-, 1.20- and 1.13-fold higher than those values of the samples harvested in 2024. The amounts of tryptophan and aspartic acid in samples harvested in 2024 were, respectively, ~2.39- and 1.44-fold higher than those of samples harvested in 2023. Subsequently, in order to find any possible differences between the two different harvest sites, the average percentages of SC-s1 and SC-s2 samples were superimposed and data are shown in Figure 1c. The resulting radar graph highlighted a similar amino acid profile between the two sites, with some quantitative differences for proline and glutamine, which are, respectively, ~2.3- and 1.6-fold higher in SC-s1 compared to SC-s2.

### 3.3. Fatty Acid Composition

In order to assess the fatty acid composition of ‘segale del Matese’ samples, lipid extracts were analyzed by GC and the obtained data are shown in Table 3. The statistical analysis did not reveal any significant differences either between the different sites or the different harvest years. Furthermore, ‘segale del Matese’ lipid extract samples contain 70% of polyunsaturated fatty acids, 18% monounsaturated fatty acids and 12% saturated fatty acids. Indeed, the percentage of unsaturated fatty acids found in the ‘segale del Matese’ samples represents about 88% of total lipids. This value is in good agreement with the literature, since it is known that rye seeds are rich in oleic, linoleic and linolenic acids, overall representing the 81.6% of the total fatty acids [5].

In particular, the most abundant fatty acids were linoleic acid and oleic acid, which represented 60% and 18% of total fatty acids, respectively. Furthermore, the remaining fatty acids (about 22% of the total fatty acids) are mainly palmitic acid and linolenic acid. These data are in agreement with Al-Taher et al. (2023), reporting a content in oleic and linoleic acids, respectively, ~1.8- and 1.1-fold lower than ‘segale del Matese’ samples, while the content of palmitic acid was ~1.3-fold higher [38].

### 3.4. Anti-Proteinase Inhibitor Activity

Antinutritional factors are secondary metabolites found in cereals and legumes that are produced by plants to protect themselves against herbivores, insects, and pathogens attacks, as well as adverse weather conditions. These compounds, including phytic acid, saponins, polyphenols, protease inhibitors, α-amylase inhibitors and lectins, can interfere with nutrient digestion and absorption in humans [39]. Indeed, protease inhibitors can reduce the proteolytic activity of pancreatic enzymes (trypsin and chymotrypsin), in turn causing a reduction in protein digestibility and amino acids absorption. In this context, soluble proteins of ‘SC-s1’ and ‘SC-s2’ rye seeds were extracted and the protease inhibitory activity of these samples was evaluated before and after the boiling process. The average amounts per year of soluble proteins from ‘SC-s1’ and ‘SC-s2’ raw rye seeds were, respectively, ~18 and 22 mg of protein extract per g of seeds; while, after boiling process, this amount was ~15-fold lower for both samples.

IC_50_ values of soluble proteins extracted from both raw and boiled seeds of ‘segale del Matese’, considering both harvest years and sites, are reported in Table 4. These data showed no significant differences between the two sites, except for the anti-tryptic activity of cooked extracts from ‘SC-s1’ and ‘SC-s2’ rye seeds. In particular, the average raw extract of ‘segale del Matese’ from both sites and years showed a higher anti-trypsin activity (IC_50_ = 8.08 μg of protein per mL of extract) with respect to anti-chymotrypsin activity (IC_50_ = 25.26 μg of protein per mL of extract).

On the other hand, the cooking process reduced the anti-trypsin activity and anti-chymotrypsin activity of about 9-fold and about 2-fold, respectively, confirming the effectiveness of heat treatment in inactivating the protease inhibitors in rye seeds. However, the presence of residual protease inhibitory activity is positive, since it is known that protease inhibitors in legumes are implicated in the suppression of carcinogenesis and the inhibition of proteolytic activities, as well as in the expression of certain proto-oncogenes in colorectal cancer [40].

### 3.5. Total Free Polyphenol Content and Antioxidant Capability of ‘Segale Del Matese’

**Figure 2:**
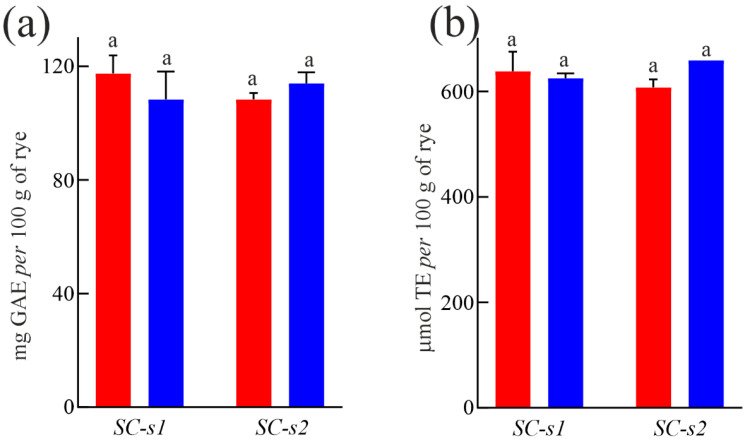
Total polyphenolic content and antioxidant capabilities of ‘segale del Matese’ collected from site 1 and 2 (SC-s1 and SC-s2, respectively) for two consecutive harvest years (2023 and 2024, shown in red and blue, respectively). (**a**) Total phenol content (TPC) and (**b**) antioxidant capacity were determined by ABTS assay. Different letters indicate statistically significant differences according to Tukey’s multiple comparisons test (*p* < 0.05).

To assess the total free polyphenol content (TPC) of ‘segale del Matese’, the SC-s1 and SC-s2 samples were subjected to ultrasound-assisted maceration using methanol/water (70:30; *v*:*v*) as the extractant (see Figure 2a). The statistical analysis did not reveal any significant differences either between the sites analyzed or the two harvest years. In particular, the average free polyphenol content of ‘segale del Matese’ was 112.02 mg GAE per 100 g seeds. This value, although comparable to the varieties analyzed by Kulichová et al. (2019) was, respectively, about 3.0-, 2.4- and 2.3-fold lower than the SVKPOL2007-40, HGP 4 and Universalne, which are the three varieties with the highest TPC [10]. These differences could be due to the extraction method used and the different soil and climate conditions.

In addition, as shown in Figure 2b, the antioxidant power of the extracts assessed using ABTS revealed no significant differences between the samples analyzed, with an average antioxidant activity of 632 µmol TE per 100 g seeds. This value was similar to that of the Niawo variety (1570 mg TE per kg; corresponding to 627 µmol TE per 100 g seeds), which is the variety with the highest antioxidant power among those analyzed by Kulichová et al. using the same antioxidant assay [10].

Overall, these findings highlight that ‘segale del Matese’ was an interesting source of natural compounds with strong antioxidant activity.

### 3.6. Tentative Identification and Quantization of the Chemical Compounds

**Figure 3:**
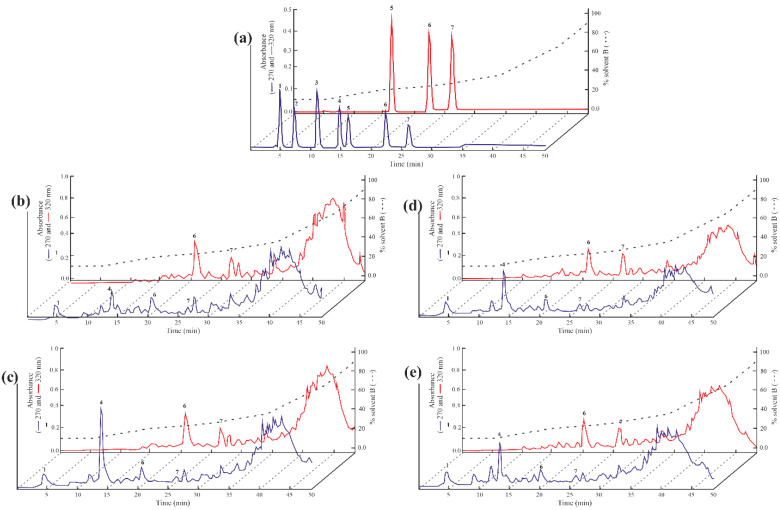
Representative RP-HPLC chromatograms of ‘segale del Matese’ free polyphenolic compound. (**a**) standard mixture. Numbers 1, 2, 3, 4, 5, 6 and 7 indicate the retention time of gallic acid, protocatechuic acid, 4-hydroxybenzoic acid, vanillic acid, caffeic acid, *p*-coumaric acid and ferulic acid, respectively. (**b**,**c**) Free polyphenolic compound of *S. cereale* site 1 (SC-s1) for two consecutive harvest years (2023 and 2024, respectively). (**d**,**e**) Free polyphenolic compound of *S. cereale* site 2 (SC-s2) for two consecutive harvest years (2023 and 2024, respectively).

The RP-HPLC analysis of free polyphenol compounds was performed according to previously published approaches [33] and the representative chromatograms in Figure 3 showed a similar qualitative profile among the different samples analyzed, with some quantitative differences.

According to the retention times, gallic acid, vanillic acid, p-coumaric acid and ferulic acid were identified in all samples and their content was reported in Table S4, showing significant differences, except for ferulic acid. Furthermore, the most abundant analyte in all samples analyzed was vanillic acid with an average value of 946 µg per 100 g seeds, followed by *p*-coumaric acid (379.88 µg per 100 g seeds), gallic acid (328.94 µg per 100 g seeds) and ferulic acid (197.04 µg per 100 g seeds). These data are in good agreement with those of 19 rye varieties analyzed by Kulichová et al. in 2019 [10]. Overall, by this approach, we obtained the partial characterization of the polyphenolic component responsible for antioxidant power from ‘segale del Matese’ methanolic extracts, within the limits of the method used.

### 3.7. Minerals Content

The mineral content of ‘segale del Matese’ is displayed in Table 5. Specifically, a comparison of the minerals between SC-s1 and SC-s2 showed significant differences only for iron, potassium and selenium. Furthermore, the data obtained revealed that potassium (485.92 mg per 100 g of rye seeds) was the most abundant mineral in terms of average mineral content, followed by phosphorus (336.67 mg per 100 g of rye seeds), magnesium (108.25 mg per 100 g of rye seeds), calcium (33.52 mg per 100 g of rye seeds). These results are in line with those previously reported for other cereals [41]. Moreover, the WHO/FAO/UNU (2007) guidelines highlight the importance of maintaining a balanced intake of sodium and potassium [42].

Indeed, both high sodium and low potassium intakes can lead to high blood pressure, which, over time, damages small blood vessels and can lead to coronary heart disease and stroke, which are major causes of death in developed countries. Therefore, the WHO recommends limiting the sodium intake and ensuring a minimum intake of potassium, by choosing foods with a high potassium to sodium (K/Na) ratio. In this context, the K/Na ratio of ‘segale del Matese’ was 231. This value was very high, similar to other flours like triticale, sorghum and barley, having K/Na ratios of 265, 177 and 107, respectively [41].

## 4. Conclusions

Rye cultivation has a long history in the Italian Apennine, where farmers selected local landraces adapted to the specific soil and climatic conditions. This selection process in Matese mountains (Campania region, southern Italy) prevented the introduction of commercial cultivars thanks to the cultivation of seeds passed down for generations. In this context, the nutritional values of ‘segale del Matese’ seeds have the potential to be exploited for the benefit of local farmers, preserving local biodiversity.

The proximate composition and biochemical traits of ’segale del Matese’ flour was characterized for the first time and the results were compared with those found in the CREA database and in the literature. In addition, data were collected by analyzing the flour obtained from ‘segale del Matese’ seeds harvested in 2023 and 2024 at both high altitudes (~1020 m a.s.l.; Castello del Matese territories; SC-s1) and low altitudes (~130 m a.s.l.; Alife territories; SC-s2).

The results show that, although few statistical differences are retrieved in the seeds grown at both altitudes, on average, ’segale del Matese’ flour had a high carbohydrate content (78.27 g per 100 g), a relatively moderate crude protein content (9.56 g per 100 g) and a low lipid content (1.27 g per 100 g) when compared to commercial rye (CREA Italian Food Database); meanwhile, its moisture content (9.32 g per 100 g) was similar to that of commercial rye (CREA). A similar trend was found when considering the total amino acid content. Indeed, for both sites SC-s1 and SC-s2, the mean values of the two years did not show significant differences, except for Glx content 1.11-fold higher than ‘SC-s2’ (altitude 130 m a.s.l.). On the other hand, the content of free amino acids (2.7% of total amino acids) showed a statistical difference with respect to both altitude and harvest year. Most of the differences were found in the proteinogenic free amino acids. In particular, asparagine and proline were found, which are involved in nitrogen transport and storage and in the response to biotic and abiotic stresses, respectively.

Moreover, in view of the antioxidant activity detected, the tentative by HPLC separation of the identification and quantification of possible metabolites able to justify this activity, highlighted the presence of gallic acid, vanillic acid, p-coumaric acid and ferulic acid in all samples (Table S4).

Finally, the mineral content of all the samples analysed was similar, as expected for iron, potassium and selenium. The K/Na ratio (231) of the ’segale del Matese’ confirms that the consumption of rye products can contribute to beneficial effects on blood pressure, contrasting cardiovascular diseases.

Thus, our study shows that the nutritional values of ‘segale del Matese’ are basically preserved in seeds grown at both high altitudes (~1020 m a.s.l.; Castello del Matese territories) and low altitudes (~130 m a.s.l.; Alife territories), making this a versatile landrace, although some significant statistical differences were observed. In addition, the antinutritional compounds of the seeds, such as trypsin and chymotrypsin inhibitors, which interfere with protein assimilation, were reduced after the boiling process.

Overall, this research on the nutritional properties of local rye landraces may have considerable benefits for mountain economy and communities, leading to the valorization of agro-ecological approach and the promotion of local food chains and agro-tourism, as well as further increasing income and diversification.

## Figures and Tables

**Table 1:** Proximate composition of ‘segale del Matese’ collected in the years 2023 and 2024 from sites 1 and 2 (SC-s1 and SC-s2, respectively), compared to commercial rye (CREA database). Values are means (±SD) of triplicate analyses (n = 3) and are expressed on dry-weight basis (g per 100 g).

	Segale del Matese		Commercial Rye
	SC-s1	SC-s2	CREA
Crude proteins	9.40 ± 0.32 a	9.73 ± 1.67 a	11.70
Lipids	1.24 ± 0.01 a	1.31 ± 0.11 a	2.00
Ash	1.77 ± 0.01 a	1.77 ± 0.01 a	-
Moisture	9.32 ± 2.33 a	9.32 ± 2.33 a	9.2
Carbohydrates	78.27 ± 2.64 a	78.27 ± 2.93 a	67.8

In each row, different letters indicate statistically significant differences according to Bonferroni’s multiple comparisons test (*p* < 0.05).

**Table 2:** Total amino acid composition of ‘segale del Matese’ collected in the years 2023 and 2024 from sites 1 and 2 (SC-s1 and SC-s2, respectively), compared to commercial rye (CREA database). Values are means (±SD) of triplicate analyses (n = 3) and are expressed on dry-weight basis (g per 100 g).

	Segale del Matese		Commercial rye
	SC-s1	SC-s2	CREA
***essential amino acids***			
His	0.19 ± 0.01 a	0.19 ± 0.04 a	0.27
Ile	0.23 ± 0.00 a	0.24 ± 0.06 a	0.49
Leu	0.50 ± 0.02 a	0.51 ± 0.10 a	0.78
Lys	0.33 ± 0.02 a	0.33 ± 0.06 a	0.48
Met	0.13 ± 0.02 a	0.14 ± 0.02 a	0.19
Phe	0.38 ± 0.01 a	0.39 ± 0.08 a	0.55
Thr	0.29 ± 0.00 a	0.30 ± 0.06 a	0.43
Trp	*n.d.*	*n.d.*	0.14
Val	0.29 ± 0.01 a	0.31 ± 0.05 a	0.61
***non-essential amino acids***			
Ala	0.38 ± 0.01 a	0.40 ± 0.09 a	0.49
Arg	0.38 ± 0.02 a	0.38 ± 0.08 a	0.57
Asx	0.47 ± 0.00 a	0.45 ± 0.10 a	0.84
Cys ^§^	0.41 ± 0.06 a	0.40 ± 0.04 a	0.23
Glx	1.61 ± 0.07 a	1.45 ± 0.08 b	2.49
Gly	0.35 ± 0.01 a	0.36 ± 0.08 a	0.56
Pro	0.79 ± 0.06 a	0.81 ± 0.28 a	0.61
Ser	0.41 ± 0.01 a	0.43 ± 0.11 a	0.49
Tyr	0.16 ± 0.02 a	0.16 ± 0.00 a	0.37
**Total**	**7.39**	**7.23**	**10.61**

Values followed by different letters indicate statistically significant differences according to Bonferroni’s multiple comparisons test (*p* < 0.05). Protein amino acids. Three-letter codes have been used: Asx—L-asparagine + L-aspartic acid; Arg—L-arginine; Cys—L-half cystine; Glx—L-glutamine + L-glutamic acid; Gly—glycine; His—L-histidine; Ile—L-isoleucine; Leu—L-leucine; Lys—L-lysine; Met—L-methionine; Phe—L-phenylalanine; Pro—L-proline; Ser—L-serine; Thr—L-threonine; Tyr—L-tyrosine; Val—L-valine. ^§^ Cys amount was evaluated after performic acid oxidation. n.d., not determined.

**Table 3:** Fatty acid constituents of ‘segale del Matese’ samples collected in the years 2023 and 2024 from sites 1 and 2 (SC-s1 and SC-s2, respectively). Values are means (±SD) of triplicate analyses (n = 3) and are expressed in terms of the percentage of oil extracted.

Fatty Acid		*‘Segale del Matese’*			
		*SC-s1*		*SC-s2*	
		2023	2024	2023	2024
*Saturated fatty acids*					
Palmitic	C16	10.35 ± 0.35 a	10.49 ± 0.46 a	11.11 ± 2.21 a	10.65 ± 0.01 a
Stearic	C18	1.12 ± 0.11 a	1.27 ± 0.03 a	1.41 ± 0.32 a	1.18 ± 0.15 a
*Monounsaturated fatty acids*					
Oleic	C18:1	16.95 ± 0.45 a	17.11 ± 1.47 a	18.08 ± 2.79 a	17.97 ± 0.54 a
*Polyunsaturated fatty acid*					
Linoleic	C18:2	59.95 ± 3.41 a	60.37 ± 3.87 a	58.96 ± 8.14 a	60.00 ± 1.72 a
Linolenic	C18:3	10.94 ± 0.66 a	10.34 ± 0.96 a	10.24 ± 1.22 a	10.40 ± 0.13 a
**SFA%**		11.47	11.76	12.52	11.82
**MUFA%**		16.95	17.11	18.08	1.18
**PUFA%**		70.90	70.72	69.20	70.40

In each row, different letters indicate statistically significant differences according to Tukey’s multiple comparisons test (*p* < 0.05).

**Table 4:** Anti-proteinase inhibitory activity of raw and cooked ‘segale del Matese’ collected in the years 2023 and 2024 from sites 1 and 2 (SC-s1 and SC-s2, respectively). Inhibitory activities obtained from the protein extracts are reported as average IC_50_ values per year and are expressed as μg of protein extract per mL.

**Anti-trypsin activity**					
***SC-s1***	2023	2024	***SC-s2***	2023	2024
*raw*	7.81 ± 0.81 a	7.86 ± 1.31 a	*raw*	9.09 ± 0.06 a	7.58 ± 1.99 a
*cooked*	67.72 ± 11.37 abc	68.32 ± 10.20 abc	*cooked*	78.98 ± 2.44 b	65.95 ± 6.40 c
**Anti-chymotrypsin activity**					
***SC-s1***	2023	2024	***SC-s2***	2023	2024
*raw*	19.56 ± 1.36 a	21.95 ± 4.06 a	*raw*	27.93 ± 7.86 a	31.59 ± 9.14 a
*cooked*	50.60 ± 2.15 a	56.78 ± 2.50 a	*cooked*	51.51 ± 3.77 a	58.26 ± 3.60 a

In each row, different letters indicate statistically significant differences according to Tukey’s multiple comparisons test (*p* < 0.05).

**Table 5:** Mineral elements of ‘segale del Matese’ collected in the years 2023 and 2024 from sites 1 and 2 (SC-s1 and SC-s2, respectively). Values are means (±SD) of triplicate analyses (n = 3) and are expressed on dry-weight basis (mg per 100 g), except Se, which is expressed on a µg/100 g of FW basis.

Element	SC-s1		SC-s2	
	*2023*	*2024*	*2023*	*2024*
Fe	3.20 ± 0.08 a	3.19 ± 0.13 a	2.62 ± 0.03 b	2.62 ± 0.04 b
Ca	35.03 ± 1.36 a	35.39 ± 1.81 a	32.33 ± 0.76 a	31.33 ± 1.02 a
Na	2.12 ± 0.10 a	2.10 ± 0.06 a	2.10 ± 0.05 a	2.10 ± 0.10 a
K	495.33 ± 15.50 a	516.67 ± 12.58 a	466.00 ± 3.61 ab	465.67 ± 12.90 b
P	333.33 ± 10.41 a	350.00 ± 10.00 a	332.33 ± 2.52 a	331.00 ± 7.94 a
Zn	2.82 ± 0.08 a	2.93 ± 0.08 a	2.77 ± 0.03 a	2.75 ± 0.05 a
Mg	110.00 ± 3.61 a	114.00 ± 4.58 a	105.00 ± 2.00 a	104.00 ± 2.00 a
Cu	0.40 ± 0.01 a	0.43 ± 0.01 a	0.40 ± 0.003 a	0.40 ± 0.01 a
Se	36.80 ± 0.26 a	36.87 ± 0.40 a	29.80 ± 0.36 b	29.07 ± 1.78 b
Mn	3.14 ± 0.20 a	3.14 ± 0.14 a	2.95 ± 0.15 a	2.93 ± 0.25 a

In each row, different letters indicate statistically significant differences according to Tukey’s multiple comparisons test (*p* < 0.05).

## Data Availability

The original contributions presented in the study are included in the article/Supplementary Material, further inquiries can be directed to the corresponding author.

## Supplementary Materials

The following supporting information can be downloaded at: https://www.mdpi.com/article/10.3390/foods14071120/s1: Table S1: Proximate composition of rye seeds from ‘segale del Matese’ collected in the years 2023 and 2024 from sites 1 and 2 (SC-s1 and SC-s2, respectively); Table S2: Total amino acid composition of rye seeds from ‘segale del Matese’, collected in the years 2023 and 2024 from sites 1 and 2 (SC-s1 and SC-s2, respectively); Table S3: Free amino acid composition of ‘segale del Matese’, collected in the years 2023 and 2024 from sites 1 and 2 (SC-s1 and SC-s2, respectively); Table S4: Contents of phenolic compounds in rye extracts from ‘segale del Matese’, collected in the years 2023 and 2024 from sites 1 and 2 (SC-s1 and SC-s2, respectively).

## References

[1] Sapirstein H.D., Bushuk W., Wrigley C., Corke H., Seetharaman K., Faubion J. (2016). Rye Grain: Its Genetics, Production, and Utilization. Encyclopedia of Food Grains.

[2] Bushuk W. (2001). Rye: Production, Chemistry, and Technology.

[3] Hannah Ritchie H., Rosado P., Roser M. Rye Production. https://ourworldindata.org/grapher/rye-production.

[4] Ahmad I., Wang H., Kamran M., Ikram K., Hou F. (2023). Simulated Grazing (Clipping) Affected Growth and Nutritional Quality of Barley, Rye, and Wheat in an Arid Climate. J. Plant Growth Regul..

[5] Ikram A., Saeed F., Noor R.A., Imran A., Afzaal M., Rasheed A., Islam F., Iqbal A., Zahoor T., Naz S. (2023). A comprehensive review on biochemical and technological properties of rye (*Secale cereale* L.). Int. J. Food Prop..

[6] Hansen H.B., Møller B., Andersen S.B., Jørgensen J.R., Hansen A. (2004). Grain characteristics, chemical composition, and functional properties of rye (*Secale cereale* L.) as influenced by genotype and harvest year. J. Agric. Food Chem..

[7] Schupfer E., Pak S.C., Wang S., Micalos P.S., Jeffries T., Ooi S.L., Golombick T., Harris G., El-Omar E. (2021). The effects and benefits of arabinoxylans on human gut microbiota—A narrative review. Food Biosci..

[8] Choromanska A., Kulbacka J., Harasym J., Dubinska-Magiera M., Saczko J. (2017). Anticancer activity of oat β-glucan in combination with electroporation on human cancer cells. Acta Pol. Pharm..

[9] Deleu L.J., Lemmens E., Redant L., Delcour J.A. (2020). The major constituents of rye (*Secale cereale* L.) flour and their role in the production of rye bread, a food product to which a multitude of health aspects are ascribed. Cereal Chem..

[10] Kulichová K., Sokol J., Nemeček P., Maliarová M., Maliar T., Havrlentová M., Kraic J. (2019). Phenolic compounds and biological activities of rye (*Secale cereale* L.) grains. Open Chem..

[11] Kaur P., Singh Sandhu K., Singh Purewal S., Kaur M., Kumar Singh S. (2021). Rye: A wonder crop with industrially important macromolecules and health benefits. Food Res. Int..

[12] Rodehutscord M., Rückert C., Maurer H.P., Schenkel H., Schipprack W., Bach Knudsen K.E., Schollenberger M., Laux M., Eklund M., Siegert W. (2016). Variation in chemical composition and physical characteristics of cereal grains from different genotypes. Arch. Anim. Nutr..

[13] Iversen K.N., Jonsson K., Landberg R. (2022). The Effect of Rye-Based Foods on Postprandial Plasma Insulin Concentration: The Rye Factor. Front. Nutr..

[14] Magnusdottir O.K., Landberg R., Gunnarsdottir I., Cloetens L., Åkesson B., Rosqvist F., Schwab U., Herzig K.H., Hukkanen J., Savolainen M.J. (2014). Whole grain rye intake, reflected by a biomarker, is associated with favorable blood lipid outcomes in subjects with the metabolic syndrome A randomized study. PLoS ONE.

[15] Larsson S.C., Giovannucci E., Bergkvist L., Wolk A. (2005). Whole grain consumption and risk of colorectal cancer: A population-based cohort of 60,000 women. Br. J. Cancer.

[16] Németh R., Tömösközi S. (2021). Rye: Current state and future trends in research and applications. Acta Aliment..

[17] Arens U., Sadler M.J. (2015). 7—Authorised EU health claim for rye fibre. Foods, Nutrients and Food Ingredients with Authorised EU Health Claims: Volume 2.

[18] Sardella C., Capo L., Adamo M., Donna M., Ravetto Enri S., Vanara F., Lonati M., Mucciarelli M., Blandino M. (2023). The cultivation of rye in marginal Alpine environments: A comparison of the agronomic, technological, health and sanitary traits of local landraces and commercial cultivars. Front. Plant Sci..

[19] Mucciarelli M., Massimo Blandino M., Capo L. (2022). La Segale in Piemonte—Storia di una Rinascita.

[20] Peratoner G., Seling S., Klotz C., Florian C., Figl U., Schmitt A.O. (2016). Variation of agronomic and qualitative traits and local adaptation of mountain landraces of winter rye (*Secale cereale* L.) from Val Venosta/Vinschgau (South Tyrol). Genet. Resour. Crop Evol..

[21] Adamo M., Blandino M., Capo L., Ravetto Enri S., Fusconi A., Lonati M., Mucciarelli M. (2021). A ddRADseq Survey of the Genetic Diversity of Rye (*Secale cereale* L.) Landraces from the Western Alps Reveals the Progressive Reduction of the Local Gene Pool. Plants.

[22] Villa T.C.C., Maxted N., Scholten M., Ford-Lloyd B. (2005). Defining and identifying crop landraces. Plant Genet. Resour..

[23] Assessorato_Agricoltura_regione_Campania Sécena. https://agricoltura.regione.campania.it/Tipici/tradizionali/secena.html.

[24] Landi N., Piccolella S., Ragucci S., Faramarzi S., Clemente A., Papa S., Pacifico S., Di Maro A. (2021). Valle Agricola Chickpeas: Nutritional Profile and Metabolomics Traits of a Typical Landrace Legume from Southern Italy. Foods.

[25] Landi N., Ragucci S., Formato M., Piccolella S., Magri A., Baiano S., Petriccione M., Papa S., Pedone P.V., Pacifico S. (2024). Wild fennel seeds from Valle Agricola (Southern Italy): Biochemical and antioxidant traits in minced pork meat. J. Funct. Foods.

[26] (2000). Official Methods of Analysis of AOAC International.

[27] (2003). Analytical methods for carbohydrates in foods. Food Energy—Methods of Analysis and Conversion Factors.

[28] Di Maro A., Dosi R., Ferrara L., Rocco M., Sepe J.J., Ferrari G., Parente A. (2011). Free Amino Acid Profile of *Malus domestica* Borkh Cv. Annurca from the Campania Region and Other Italian Vegetables. Aust. J. Crop Sci..

[29] Moore S., Stein W.H. (1963). Chromatographic determination of amino acids by the use of automatic recording equipment. Methods in Enzymology.

[30] Landi N., Alberico L., Clemente A., Peddio S., Hussain H.Z.F., Ragucci S., Zucca P., Woodrow P., Di Maro A. (2023). Nutritional, metabolic and genetic profiling of ‘Cerato’ and ‘Curniciello’ bean landraces from Caserta, Southern Italy. Food Biosci..

[31] Poerio E., Gennaro S.D., Di Maro A., Farisei F., Ferranti P., Parente A. (2003). Primary Structure and Reactive Site of a Novel Wheat Proteinase Inhibitor of Subtilisin and Chymotrypsin. Biol. Chem..

[32] Landi N., Pacifico S., Piccolella S., Di Giuseppe A.M., Mezzacapo M.C., Ragucci S., Iannuzzi F., Zarrelli A., Di Maro A. (2015). Valle Agricola lentil, an unknown lentil (*Lens culinaris* Medik.) seed from Southern Italy as a novel antioxidant and prebiotic source. Food Funct..

[33] Irakli M.N., Samanidou V.F., Biliaderis C.G., Papadoyannis I.N. (2012). Development and validation of an HPLC-method for determination of free and bound phenolic acids in cereals after solid-phase extraction. Food Chem..

[34] Landi N., Ragucci S., Fiorentino M., Guida V., Maro A.D. (2017). Nutritional values and metabolic profile with and without boiled treatment of ’Gallo Matese’ beans (*Phaseolus vulgaris* L.), a landrace from Southern Italy. Acta Sci. Pol. Technol. Aliment..

[35] AlimentiNUTrizione—Ricerca Dati. https://www.alimentinutrizione.it/tabelle-nutrizionali.

[36] Postles J., Curtis T.Y., Powers S.J., Elmore J.S., Mottram D.S., Halford N.G. (2016). Changes in Free Amino Acid Concentration in Rye Grain in Response to Nitrogen and Sulfur Availability, and Expression Analysis of Genes Involved in Asparagine Metabolism. Front. Plant Sci..

[37] Curtis T.Y., Powers S.J., Balagiannis D., Elmore J.S., Mottram D.S., Parry M.A., Rakszegi M., Bedö Z., Shewry P.R., Halford N.G. (2010). Free amino acids and sugars in rye grain: Implications for acrylamide formation. J. Agric. Food Chem..

[38] Al-Taher F., Nemzer B. (2023). Effect of Germination on Fatty Acid Composition in Cereal Grains. Foods.

[39] Salim R., Nehvi I.B., Mir R.A., Tyagi A., Ali S., Bhat O.M. (2023). A review on anti-nutritional factors: Unraveling the natural gateways to human health. Front. Nutr..

[40] Lima A.I.G., Mota J., Monteiro S.A.V.S., Ferreira R.M.S.B. (2016). Legume seeds and colorectal cancer revisited: Protease inhibitors reduce MMP-9 activity and colon cancer cell migration. Food Chem..

[41] Torbica A., Belović M., Popović L., Čakarević J., Jovičić M., Pavličević J. (2021). Comparative study of nutritional and technological quality aspects of minor cereals. J. Food Sci. Technol..

[42] WHO (2007). Joint WHO/FAO/UNU Expert Consultation. Protein and Amino Acid Requirements in Human Nutrition.

