# Supplementary material for: Nutritional Values and Biochemical Traits of Rye (Secale cereale L.) Seeds, a Landrace from Matese Mountains (Southern Italy)
Source: Foods. 2025 Mar 24;14(7):1120. doi: 10.3390/foods14071120 (PMC11988677; doi:10.3390/foods14071120)
Supplement: Supplementary file 1 [file foods-14-01120-s001.zip › foods-3530625-supplementary.pdf]

SUPPLEMENTARY MATERIALS

Nutritional values and biochemical traits of rye (*Secale cereale* L.) seeds, a landrace from Matese mountains (Southern Italy)

Nicola Landi [1, 2,*], Sara Ragucci [2], Maria Giuseppina Campanile [2], Hafiza Z. F. Hussain [2], Stefania Papa [2], Antimo Di Maro [2, *]

[1] Institute of Crystallography, National Research Council, Via Vivaldi 43, 81100-Caserta, Italy
[2] Department of Environmental, Biological and Pharmaceutical Sciences and Technologies (DiSTABiF), University of Campania 'Luigi Vanvitelli', Via Vivaldi 43, 81100 Caserta, Italy

* Co-correspondence: nicola.landi@cnr.it (NL) and antimo.dimaro@unicampania.it (ADM)

Table S1. Proximate composition of rye seeds from 'segale del Matese' collected in the years 2023 and 2024 from sites 1 and 2 (SC-s1 and SC-s2, respectively). Values are means (±SD) of triplicate analyses (n = 3) and are expressed on dry-weight basis (g per 100 g).

	**SC-s1**		**SC-s2**	
	2023	*2024*	*2023*	*2024*
Crude proteins	9.62±0.05a	9.17±0.04b	10.91±0.28c	8.55±0.10d
Lipids	1.23±0.06a	1.25±0.08a	1.39±0.20a	1.23±0.03a
Ash	1.78±0.03a	1.77±0.02a	1.89±0.13a	1.86±0.02a
Moisture	10.97±0.00a	7.68±0.39b	9.90±0.11c	8.31±0.21d
Carbohydrates	76.40±0.04a	80.14±0.24b	75.91±0.23c	80.05±0.37b

In each row, different letters indicate statistically significant differences according to Tukey's multiple comparisons test ($p<0.05$).

Table S2. Total amino acid composition of rye seeds from 'segale del Matese', collected in the years 2023 and 2024 from sites 1 and 2 (SC-s1 and SC-s2, respectively). Values are means (±SD) of triplicate analyses (n = 3) and are expressed on dry-weight basis (g per 100 g).

	**SC-s1**		**SC-s2**	
	2023	*2024*	*2023*	*2024*
essential amino acids				
His	0.18±0.02a	0.19±0.00a	0.22±0.04a	0.17±0.00a
Ile	0.23±0.01a	0.23±0.01a	0.28±0.06a	0.19±0.00a
Leu	0.49±0.01ab	0.52±0.02ab	0.58±0.14b	0.43±0.03a
Lys	0.32±0.03a	0.35±0.00a	0.37±0.05a	0.28±0.00a
Met	0.12±0.01a	0.15±0.00a	0.15±0.02a	0.12±0.01a
Phe	0.37±0.01a	0.39±0.01a	0.45±0.07a	0.33±0.01a
Thr	0.29±0.02ab	0.29±0.00ab	0.35±0.03b	0.26±0.00a
Trp	*n.d.*	*n.d.*	*n.d.*	*n.d.*
Val	0.29±0.02a	0.30±0.00a	0.34±0.05a	0.27±0.01a
non-essential amino acids				
Ala	0.38±0.03ab	0.39±0.01ab	0.46±0.09b	0.33±0.02a
Arg	0.36±0.05a	0.39±0.03a	0.43±0.10a	0.32±0.01a
Asx	0.47±0.04ab	0.47±0.01ab	0.52±0.09b	0.38±0.01a
Cys[§]	0.37±0.11a	0.46±0.02a	0.43±0.02a	0.37±0.05a
Glx	1.63±0.08a	1.73±0.04a	1.50±0.02b	1.39±0.01b
Gly	0.34±0.02a	0.36±0.00a	0.41±0.09a	0.30±0.01a
Pro	0.84±0.01a	0.75±0.04a	1.01±0.14b	0.61±0.01c
Ser	0.42±0.07ab	0.40±0.04ab	0.51±0.10b	0.36±0.01a
Tyr	0.14±0.00a	0.17±0.01a	0.16±0.04a	0.16±0.03a
**Total**	**7.24**	**7.53**	**8.17**	**6.30**

In each row, different letters indicate statistically significant differences according to Tukey's multiple comparisons test ($p<0.05$). *n.d.*, not determined.[§], Cys amount was evaluated after performic acid oxidation.

Table S3. Free amino acid composition of 'segale del Matese', collected in the years 2023 and 2024 from sites 1 and 2 (SC-s1 and SC-s2, respectively). Values are means (±SD) of triplicate analyses (n = 3) and are expressed on dry-weight basis (mg per 100 g).

	**SC-s1**		**SC-s2**	
	2023	*2024*	*2023*	*2024*
proteinogenic free amino acids				
Ala	14.69±0.80ab	16.75±0.65a	15.11±1.79a	12.26±1.21b
Arg	8.10±0.27ab	9.31±0.70ab	10.15±0.83a	6.54±1.30b
Asn	52.14±2.32a	75.29±2.82ab	52.48±8.41b	55.76±7.53ab
Asp	4.69±0.16a	7.92±0.63b	4.94±0.65a	6.32±0.94ab
Cys	0.16±0.01a	0.64±0.19a	0.16±0.01a	0.50±0.12a
Gln	15.47±4.33ad	25.34±2.62bd	7.61±2.27c	5.97±0.92cd
Glu	16.93±0.48a	22.12±1.57b	15.37±1.82ac	13.27±1.94c
Gly	4.07±0.24a	4.16±0.08a	4.05±0.34a	3.27±0.37a
His	1.49±0.09a	2.41±0.02a	2.29±0.11a	1.93±0.30a
Ile	4.43±0.11a	4.08±0.01a	3.97±0.34a	3.00±0.26a
Leu	4.55±0.25a	4.22±0.10a	4.08±0.42a	3.21±0.29a
Lys	2.48±0.17a	2.39±0.01a	2.35±0.14a	1.62±0.21a
Met	1.60±0.12a	1.38±0.17a	1.30±0.15a	0.63±0.17a
Phe	2.74±0.16a	3.53±0.07a	2.80±0.28a	2.67±0.19a
Pro	21.60±1.34a	14.99±0.13b	10.77±1.44c	7.18±0.73d
Ser	5.72±0.44a	5.57±0.26a	5.22±0.49a	3.43±0.24a
Thr	5.30±0.32a	5.25±0.20a	5.14±0.58a	3.69±0.38a
Trp	1.80±0.16a	3.00±0.09ab	2.55±0.45ab	5.43±0.58b
Tyr	2.48±0.09a	3.65±0.23a	3.34±0.49a	2.96±0.49a
Val	9.08±1.22a	8.04±0.14ab	7.30±0.74ab	5.40±0.46b
non-proteinogenic free amino acids				
AAAA	1.47±0.50a	2.05±0.15a	1.47±0.54a	1.347±0.004a
β-ala	1.11±0.06a	0.98±0.23a	1.13±0.06a	0.54±0.08a
Ethan	2.03±0.05a	1.73±0.28a	2.21±0.21a	1.49±0.06a
Gaba	4.26±0.29a	3.73±0.51a	5.270±0.004a	4.29±0.64a
Pea	0.86±0.21a	1.27±0.25a	1.28±0.10a	0.91±0.17a
Taur	0.91±0.04a	1.00±0.11a	1.08±0.17a	1.00±0.06a
**Total**	**190.17**	**230.80**	**173.81**	**154.63**

In each row, different letters indicate statistically significant differences according to Tukey's multiple comparisons test ($p<0.05$). AAAA, β-ala, Ethan, Pea, Gaba and Taur were α-aminoadipic acid, β-alanina, ethanolamine, γ-aminobutyric acid, phosphorylethanolamine and taurine, respectively.

Table S4. Contents of phenolic compounds in rye extracts from 'segale del Matese', collected in the years 2023 and 2024 from sites 1 and 2 (SC-s1 and SC-s2, respectively). Values are means (±SD) of triplicate analyses (n = 3) and are expressed on dry-weight basis (µg per 100g of rye seed).

Compound	**SC-s1**		**SC-s2**	
	2023	*2024*	*2023*	*2024*
gallic acid	408.66±38.37a	324.19±22.94ab	293.12±15.94b	289.78±20.89b
vanillic acid	441.02±32.45a	1812.57±98.35b	810.27±23.90c	712.69±9.96c
p-coumaric acid	472.60±60.92a	386.01±10.36ab	307.82±7.96b	353.08±18.87b
ferulic acid	158.44±51.48a	205.30±87.90a	224.74±35.87a	199.67±35.09a

In each row, different letters indicate statistically significant differences according to Tukey's multiple comparisons test ($p<0.05$).
